# Supplementary material for: A master protocol to investigate a novel therapy acetyl-l-leucine for three ultra-rare neurodegenerative diseases: Niemann-Pick type C, the GM2 gangliosidoses, and ataxia telangiectasia
Source: Trials. 2021 Jan 22;22:84. doi: 10.1186/s13063-020-05009-3 (PMC7821839; doi:10.1186/s13063-020-05009-3)
Supplement: Supplementary file 1 — Additional file 1: Supplementary Material I. Video Acquisition, Submission, and Review. Supplementary Material II. Safety Parameters. Supplementary Material III. Data Collection. [file 13063_2020_5009_MOESM1_ESM.docx]

**Supplementary Material I: Video Acquisition, Submission, and Review**

The Medpace Core Laboratory is contracted to manage the collection, quality control, central review, and storage of the subject video data acquired for the IB1001 studies. Medpace Core Laboratory provides a video acquisition manual to each clinical site, as well as a GoPro 7 (recorder), a kit of camera accessories, and a tripod. All videos should be recorded using the equipment and according to the acquisition manual provided by Medpace Core Laboratory to ensure consistency and minimize variability.

There are no specific qualifications for the designated clinical site videographer(s). Any individual at the site willing to undergo video acquisition, export, and upload training will be eligible. Each potential videographer is asked to submit two qualification "sample" videos for the purpose of qualification. The videos should be of a volunteer performing the 8MWT and 9HPT-D, acquired using the instructions outlined in a video acquisition manual. Informed consent from the volunteer(s) is obtained (if applicable). The qualification sample video submissions will confirm the ability of the clinical site to acquire and export videos in the correct file format and electronically submit to Medpace Core Laboratory.

Video informed consent from each patient will be acquired. Videos will be collected by the clinical sites and submitted to Medpace Core Laboratory for collection, quality control, central review, and storage. All patient videos should be performed by the same videographer if possible, to ensure consistency and reduce the potential for variability. All study-related video files are submitted to Medpace Core Laboratory via the web-based ClinTrak Imaging Windows Client Application. All submitted videos undergo QC (of the submitted transmittal form and storage in the ClinTrak Imaging system) within two business days of receipt by Medpace Core Laboratory. If a video is of unacceptable quality during the quality assessment, Medpace Core Laboratory may request a repeat video which should be performed as soon as possible, assuming this is feasible as determined by the investigator. Even if the video cannot be repeated, the purpose of the quality assessment is to identify issues and prevent repetition of similar quality issues on subsequent videos.

**Supplementary Material II: Safety Parameters**

**Laboratory examinations - blood**

A routine blood sample will be taken to exclude liver or kidney failure, and a pregnancy test for women of childbearing potential will be performed. The following laboratory parameters will be assessed at each visit (except Visit 0) via blood draw: hemoglobin, erythrocytes, hematocrit, thrombocytes, leukocytes, sodium, lactate dehydrogenase, potassium, creatinine, serum bilirubin level, aspartate aminotransferase, alanine aminotransferase, urea, alkaline phosphatase, follicle-stimulating hormone (for postmenopausal women only). Rum bicarbonate, chloride, calcium and phosphorus will also be assessed at each visit (except Visit 0) for the IB1001-203 study.

Sparse Pharmacokinetic blood sampling will be conducted during the initial treatment sequence (Visit 1 to Visit 6) at every visit. Blood samples for the quantification of N-acetyl-L-leucine in plasma will be obtained at Visit 7 and Visit 9. The first sample will be taken before first /last dosing of N-acetyl-L-leucine in the Extension Phase; subsequent samples will be taken at 30 minutes (+/- 5 minutes), 60 minutes (+/- 5 minutes), 90 minutes (+/- 10 minutes), 120 minutes (+/- 10 minutes), 150 (+/- 15 minutes), 180 minutes (+/- 15 minutes), 240 minutes (+/- 15 minutes), and 360 minutes (+/- 15 minutes) after the first/last extension phase IB1001 dose.

The total amount of blood taken per subject during the first treatment sequence (Visit 1 – Visit 6) will be 66 mL (42 mL blood for the safety analyses, 24 mL blood for the Pharmacokinetic analyses). The total amount of blood taken per subject during the Extension phase treatment sequence is 85 mL – 92 mL (21 mL – 28 mL (depending on the date of Visit 7) for safety analysis, and 64 mL for the Pharmacokinetic analysis.

**Laboratory examinations – urine**

The following laboratory parameters will be assessed via urine at each visit (except Visit 0): Leukocytes, Nitrite, Urobilinogen, Protein, pH, Occult blood (erythrocytes, leucocytes), Specific gravity, Ketones, Bilirubin, Glucose.

Urine samples will also be collected for concentrations of N-acetyl-D-leucine at the time points designated on the schedule of events. At Visit 1, this Urine sample score serves as a key enrollment criterion testing for the use of the prohibited medication N-acetyl-DL-leucine.

**Height, Weight, Vital Signs, Physical Exam**

Patient’s height, weight, systolic/diastolic blood pressure and pulse will be measured routinely throughout the duration of the study according to the schedule of events (Suppl. Table 1).

A complete physical examination that is limited to the following body systems will be conducted according to the schedule of events (Suppl. Table 1): general appearance (including skin), head and neck, eyes and ears, nose and throat, pulmonary, cardiovascular, gastrointestinal, musculoskeletal, lymphatic, and neurological.

### Electrocardiograms

12-lead electrocardiograms will be performed at the time points designated on the schedule of events (Suppl. Table 1) and include the following measurements: Corrected QT, and whether the electrocardiograms is Normal, Abnormal not clinically significant, or Abnormal clinically significant for the patient. Intervals of the electrocardiograms will be recorded in the electronic case report form. Electrocardiograms may be performed at other visits at the discretion of the Investigator.

**Concomitant drug and non-drug therapies**

Trial participants should not begin physiotherapy or speech therapy while they are enrolled in the trial. If they are already under therapy, the number of sessions of physiotherapy and speech therapy (measured in hours of therapy per week) will be documented in the patient’s medical record and in the electronic case report form during the trial and for 6 weeks prior to screening. To adhere to the protocol, the non-pharmacological concomitant therapy should be consistent 6-weeks before Visit 1 and continued with the same intensity while the patient is enrolled in the trial.

Guided by the eligibility criteria, the administration of aminopyridines, Riluzole, Varenicline, Gabapentin or Chlorzoxazone is not allowed during the trial because of a possible beneficial effect in Cerebellar Ataxia. Variants of the IMP, -Acetyl-DL-Leucine (e.g. Tanganil^®^) or N-acetyl-L-leucine (if not provided as IMP) are not allowed during the trial. Sulfasalazine and Rosuvastatin are not allowed because drug interactions with substrates of transporters MDR1, BCRP, or BSEP cannot be excluded as N-acetyl-L-leucine is an inhibitor of these transporters *in vitro.*

**Participation discontinuation**

Patients may withdraw from the study at any time at their own request without stating the reason(s) for withdrawal. The Principal Investigator may decide that a patient should be withdrawn from the study or from the study drug. Patients who discontinue study drug prior to completing the full treatment period will be asked to complete the remaining study visits as far as possible and complete safety assessments at a minimum. If unwilling to complete the remaining study visits, regardless of the reason for withdrawal, best efforts should be made to have the patient take part in early termination procedures, preferably 7-14 days following the last dose of the study drug, unless the patient is lost to follow-up or has withdrawn his/her consent to further study participation. The reason for withdrawal (if available) will be recorded in the electronic case report form.

**Safety assessment**

The Principal Investigator is responsible for monitoring the safety of patients who have been enrolled in this study and for accurately documenting and reporting information as described in this section.

In addition, the investigator will monitor the degree of stress to patients and the risk threshold throughout the trial.

Patients will be instructed to report to the Principal Investigator any Adverse Events that they experience. The Principal Investigator will ask about the occurrence of Adverse Events at each visit. Investigators are required to document all Adverse Events occurring during the clinical study, commencing with the signing of the informed consent form through the End of Study Visit (scheduled at 42 days post last IB1001 dose). Adverse event recording will continue for patients who discontinue study treatment but remain on-study, until their early termination Visit has been completed. The Principal Investigator will judge the intensity (mild, moderate, or severe), seriousness, and causality (not related or related) of all adverse events.

All adverse events will be listed by trial site and patient and displayed in summary tables that provide data combined across all sites. The incidence of adverse events and their relationship to the study drug will be analyzed descriptively, guided by the Medical Dictionary for Regulatory Activities classification.

**Supplementary Material III: Data collection**

Study data for all patients will be collected in a confidential fashion using an electronic case report form supported by Viedoc. Access to the electronic case report form is restricted to staff members not involved in any aspect of the blinded evaluations. All the information required by the protocol must be documented and any omissions explained. The Investigator must review all electronic case report form entries for completeness and accuracy. Source documents, including all demographic and medical information, electronic case report form and informed consent form for each patient in the study must be maintained by the Investigator. All information in the electronic case report form must be traceable to the original source documents. An audit trail of all changes to this database, including the date, reason for the data change and who made the change, will be maintained within the same database. The audit trail will be part of the archived data at the end of the study. Concomitant medication and adverse events will be coded using standardized medical dictionaries.
