# Supplementary material for: A master protocol to investigate a novel therapy acetyl-l-leucine for three ultra-rare neurodegenerative diseases: Niemann-Pick type C, the GM2 gangliosidoses, and ataxia telangiectasia
Source: Trials. 2021 Jan 22;22:84. doi: 10.1186/s13063-020-05009-3 (PMC7821839; doi:10.1186/s13063-020-05009-3)
Supplement: Supplementary file 2 — Additional file 2: Supplementary Table 1. Parent Study schedule of enrolment, interventions, and assessments. Supplementary Table 2. Extension Phase schedule of enrolment, interventions, and assessments. [file 13063_2020_5009_MOESM2_ESM.docx]

**Supplementary Table 1**

|  | *Non-naïve patients only* | *Non-naïve patients only* |  |  |  |  |  |  |  |
| --- | --- | --- | --- | --- | --- | --- | --- | --- | --- |
| Patient information and informed consent process | X |  | X^1^ |  |  |  |  |  |  |
| Inclusion / exclusion criteria | X |  | X | X |  |  |  |  |  |
| Patient weight and height measurements | X |  | X^1^ |  |  |  |  |  |  |
| Physical Exam (203 study only) |  |  |  |  |  |  |  |  |  |
| Vital signs | X |  | X | X | X | X | X | X | X |
| Patient demographics (in accordance with local regulations) | X |  | X^1^ |  |  |  |  |  |  |
| Relevant medical history | X |  | X^1^ |  |  |  |  |  |  |
| 60-Day drug history | X |  | X^1^ |  |  |  |  |  |  |
| Documentation of concomitant medication | X |  | X | X | X | X | X | X | X |
| Documentation of therapy | X |  | X | X | X | X | X | X | X |
| Confirmation prohibited medications have not been used in the past 42 days at Visit 1/since Visit 1 for Visit 2 |  |  | X | X |  |  |  |  |  |
| Confirmation prohibited medications have been used within past 42 days | X |  |  |  |  |  |  |  |  |
| Classify patient as “Naïve” or “Non-naïve” | X |  | X^1^ |  |  |  |  |  |  |
| 12-lead electrocardiogram |  |  | X |  | X |  | X |  | X |
| Urine Test for N-Acetyl-D-Leucine |  |  | X | X |  |  | X | X | X |
| Urine by dipstick for pregnancy test (if applicable) |  |  | X^2^ | X |  | X |  | X | X |
| Urinalysis |  |  | X | X | X | X | X | X | X |
| Blood safety laboratory tests |  |  | X | X | X | X | X | X | X |
| Blood sample for sparse PK |  |  | X | X | X | X | X | X | X |
| Quality of Life EQ-5D-5L for patients aged ≥18 years; EQ-5D-Y for children aged <18 years |  |  | X | X | X | X | X | X | X |
| Scale for Ataxia Rating (SARA) | X |  | X | X | X | X | X | X | X |
| Scale for Spinocerebellar Ataxia Functional Index (SCAFI) | X |  | X | X | X | X | X | X | X |
| Cognitive assessment according to standard procedures of the clinical site |  |  | X |  |  |  |  |  |  |
| Determination of CI-CS Primary Anchor Test (9HPT-D or 8MWT) |  |  | X |  |  |  |  |  |  |
| Modified Disabling Rating Score (201 and 202 study only) |  |  | X | X | X | X | X | X | X |
| Niemann-Pick Disease type C Clinical Severity Scale (201 study only) |  |  | X |  |  |  |  |  |  |
| Columbia Suicide Severity Rating Scale (203 study only) | X |  | X | X | X | X | X | X | X |
| Clinical Global Impression of Severity Physician, Caregiver, Patient (if able) |  |  | X | X | X | X | X | X | X |
| Clinical Global Impression of Improvement by Physician, Caregiver, Patient (if able) |  |  |  |  |  | X |  | X | X |

| Documentation of adverse events | X |  | X | X | X | X | X | X | X |
| --- | --- | --- | --- | --- | --- | --- | --- | --- | --- |
| Dispensing of study drug |  |  |  | X | X |  |  |  |  |
| Intake of study drug at site |  |  |  | X |  |  |  |  |  |
| Return of study drug |  |  |  |  | X | X |  |  | X |
| Study drug compliance check |  |  |  |  | X | X |  |  | X |

^1 “^Naïve” patient only

^2^ IB1001-203 only
